# Seasonal variations in urinary calcium, volume, and vitamin D in kidney stone formers

**DOI:** 10.1590/S1677-5538.IBJU.2018.0095

**Published:** 2018

**Authors:** Kyrollis Attalla, Shubha De, Carl Sarkissian, Manoj Monga

**Affiliations:** 1 Glickman Urologic & Kidney Institute, Cleveland Clinic, OH, USA

**Keywords:** Kidney, Calculi, Urinary Tract

## Abstract

**Objectives:**

To investigate the seasonal variations in urinary calcium, serum vitamin D, and urinary volume in patients with a history of nephrolithiasis.

**Materials and Methods:**

Patients included were those who completed a 24-hour urine metabolic evaluation on two occasions; one in summer (June-Aug) and one in winter (Nov-Jan), and who had not started any medications or been instructed on dietary modifications in the interval between the two tests that may have impacted the results. Patients were excluded if they were on thiazide diuretics or were taking calcium and / or Vitamin D supplementation. Welch's t-test was used to compare the difference in average summer and winter values. Unpaired Student t-test was used to compare baseline parameters (age, BMI), and Paired Student t-test was used to compare average seasonal measurements in men vs. women.

**Results:**

136 patients were identified who were not taking calcium or vitamin D supplements or thiazide diuretics, and who were not instructed on dietary modifications in the interval between the two measured parameters. No significant differences were observed when comparing male to female baseline parameters of age or BMI (Table-1). Average 24-hour urine calcium was higher (226.60) in the winter than in summer (194.18) and was significant in males (p = 0.014) and females (p < 0.001). No significant seasonal difference was seen in 24-hour urine volume or serum vitamin D levels.

**Conclusions:**

Urinary calcium is higher in winter months compared to summer months. As such, tailoring medical preventative strategies to the time of year may be helpful.

## INTRODUCTION

Kidney stone disease is largely attributable to several metabolic risk factors, including urinary calcium excretion and hydration status. The pathophysiologic role of vitamin D status in stone disease remains controversial (1–3). The seasonal variation in stone risk factors is a salient issue, as these variations have implications for preventive therapy. Several studies assert that vitamin D levels are influenced by season (4), but seasonal variations in vitamin D status have not been studied specifically as it relates to kidney stone disease.

Approximately 80% of stones are predominantly calcium based: calcium oxalate (70%) or calcium phosphate (10%) (5). High urine calcium is the most common abnormality seen in recurrent stone formers after low urine volume, and since factors like vitamin D potentially vary by season, so may urinary calcium excretion. The objective of this study was thus to assess whether vitamin D levels and urinary parameters, namely calcium and volume, vary between summer and winter months in stone formers.

## MATERIALS AND METHODS

A retrospective search of our patient registry for patients with 24-hour urine calcium levels in both summer and winter spanning 10 years (2003-2013) was conducted. Data was collected in an Institutional Board Review (IRB) approved protocol. Patient characteristics regarding age and body mass index (BMI) were obtained. Summer months were considered to be June, July, and August, and the winter months were November, December, and January. Patients were included if the time between summer and winter 24-hour urine calcium was 6-18 months. Summer and winter 24-hour urine volume was also recorded. Patients were included only if they were not taking supplements (calcium or vitamin D) or thiazide diuretics. All patients included were seen in a stone clinic and have a documented history of nephrolithiasis.

Summer and winter vitamin D (measured by chemiluminescence immunoassay ng / dL) levels were also recorded for each patient if they were performed within 3 months of each 24-hour urine composition. Unpaired Student t-test was used to compare baseline parameters between men and women, including age and BMI. Paired Student t-test was used to compare the means of summer and winter measurements in men, women, and for the total group, and Welch's t-test was used to compare the delta in men versus women, defined as the difference in mean summer values minus mean winter values in both groups. A p-value < 0.05 was considered to be statistically significant.

## RESULTS

A total of 136 patients with a history of nephrolithiasis were identified, 85 males and 51 females, who were not taking calcium or vita-min D supplements or thiazide diuretics, and who were not instructed on dietary modifications in the interval between the two measured parameters. No significant differences were observed when comparing male to female baseline parameters of age or BMI (Table-1). It is noteworthy to mention that both males and females are classified as “overweight,” or “pre-obese” as per the World Health Organization (WHO) according to their BMI. Our cohort of patients showed higher urinary calcium levels in the winter (226.60 mg / day winter vs. 194.18 mg/day summer, p < 0.001) (Table-2). Serum vitamin D and 24-hour urine volumes failed to show significant seasonal variations, and gender did not appear to impact the differences in in seasonal urinary calcium, serum vitamin D, or 24-hour urine volume.

**Table 1:** Patient Characteristics.

	Males	Females	p-value	Total
Patients	85	51	-	136
Mean age (years)	50.94 ± 15.24	47.57 ± 17.11	p = 0.89	50.79 ± 15.89
Mean BMI	29.59 ± 8.46	29.04 ± 6.38	p = 0.70	29.38 ± 7.71

**Table 2:** Seasonal Variations (mean).

		Males	*p*-value	Females	*p*-value	Males vs. Females	*p*-value	Males and Females	*p*-value
24-HOUR URINE CALCIUM									
**Urine calcium**									
	Summer	205.72	**0.014**	176.65	**<0.001**	--	0.216	194.18	**<0.001**
	Winter	233.03		216.84		--		226.60	
SERUM VITAMIN D									
**Vitamin D**									
	Summer	27.14	0.182	37.29	0.312	--	0.386	33.11	0.169
	Winter	32.64		40.35		--		37.18	
24-HOUR URINE VOLUME									
**Mean volume**									
	Summer	2134.26	0.266	2171.75	0.202	--	0.359	2148.77	0.231
	Winter	2039.06		2059.99		--		2069.48	

## DISCUSSION

Patients with nephrolithiasis have a propensity for increased intestinal calcium absorption, urinary calcium excretion, and bone mineral loss, and vitamin D has been implicated in all of these processes (6). Although the role of vitamin D in nephrolithiasis remains controversial, serum vitamin D levels have been shown to generally decrease in winter months compared to summer months due to reduced exposure to sunlight (7). The relationship between serum vitamin D and urinary calcium excretion is ill-defined in our study we demonstrated that despite non-significant differences in serum vitamin D, urinary calcium excretion was found to be significantly greater in winter months compared to summer months, a finding supported by prior studies (8).

Several investigations report no difference in serum vitamin D levels between hypercalciuric and normocalciuric stone formers (1, 2, 9). Though other studies suggest a positive correlation between vitamin D and calcium excretion, this was not identified in our patient population (3, 10). A study of healthy postmenopausal women found that vitamin D supplementation did not increase urinary calcium excretion (11). Similarly, Leaf et al. found vitamin D levels to increase significantly after repletion, however no effect was seen on 24-hour urinary calcium (12).

The majority of our patients were either classified as overweight or obese according to their body mass index. BMI has been found to be associated with increasing urinary calcium (13–15), urinary uric acid supersaturation (16), and calcium oxalate stone formation (17). Similarly, increasing BMI has been demonstrated to inversely correlate with serum vitamin D levels (18, 19). As such, trends in serum levels seen in non-obese populations may have been muted in our analysis.

Seasonal physiologic and behavioral changes may help explain some of the effects on urinary indices, vitamin D levels, and resultant stone risk. Environmental factors related seasonal variations may impact people with kidney stones. UV exposure, humidity, and activity patterns have all been hypothesized to play a role in stone formation (20). Previously, studies assessing seasonal urine volumes and stone risk have reported heterogeneous findings. It has been shown that warmer temperatures in the summer months reduce urine volume, thereby increasing the risk of nephrolithiasis (21). Other studies support our findings that urine volume does not differ across seasons (22, 23).

Dietary patterns may also change based on the availability of fresh produce, and a reliance on packaged / processed foods in colder seasons. Though this would suggest an increase in urinary sodium, and subsequently calcium, the latter was seen in our study. As UV exposure is reduced in winter months, dietary vitamin D (i.e. cod liver oil, fish, fortified cereals, etc.) becomes a significant contributor, with significant variations based on ethnicity (24). We did not evaluate seasonal variations in dietary intake.

Seasonal weight fluctuations may again add to the complexity of the relationships. Activity levels are known to be reduced in winter months (25), and coinciding North American holidays (i.e. Thanksgiving, Christmas, New Year) are associated with indulgent consumption. If these factors lead to relative weight gain in colder months, changes in urinary calcium may follow. Lower baseline vitamin D levels have also been associated with increased weight gains in a prospective longitudinal study of 4660 women > 65 years old (26).

Winter has also been associated with peaks in myocardial infarction, sudden cardiac death, stroke / TIA, pulmonary embolisms and congestive heart failure (27). A cohort study of over 3 million health claims from Canada has identified a strong association between kidney stones and cardiovascular events, suggesting a common pathophysiology affecting both processes (28). Elevated CRP levels are a well-known risk factor for cardiovascular events, and have also been associated with kidney stones (29). These levels show a seasonal variation with peaks noted in winter months (30), suggesting a common inflammatory mechanism in certain patients.

Several limitations exist in our retrospective study. As diet and hydration status may vary between seasons and play critical roles in stone disease, we were unable to account for these factors. Although all patients included in the study are from the same institution, high-volume centers may receive patients who do not reside in the same city or state. No demographic data regarding geographic location of patients, climate, sunlight exposure, and time spent outdoors, were available for these patients. Though the majority of our patients are from mid-western United States, we cannot comment specifically on their location at the time of lab / urine investigations. Specifically, it would be interesting to evaluate seasonal changes in urinary calcium levels in those who work indoors at a set ambient temperature with little sunlight exposure to those who work outdoors. Finally, given our limited numbers, trends may exist which we were unable to capture. The clinical implications of our study suggest that preventive strategies for patients with hypercalciuria could be tailored to the time of year.

## CONCLUSIONS

Urinary calcium excretion was demonstrated to be significantly higher in winter months compared to summer months in both males and females. No seasonal differences with respect to serum vitamin D and 24-hour urine volume were observed. These findings provide a better understanding of the propensities in stone risk factors, and may stimulate more targeted stone prevention strategies that could be varied based on the season.
